# Supplementary material for: COVID-19 and Intracranial Hemorrhage: A Multicenter Case Series, Systematic Review and Pooled Analysis
Source: J Clin Med. 2022 Jan 25;11(3):605. doi: 10.3390/jcm11030605 (PMC8836638; doi:10.3390/jcm11030605)
Supplement: Supplementary file 1 [file jcm-11-00605-s001.zip › Supplementary File S1.pdf]

Supplemental 1

((cerebral hemorrhage[MeSH Terms]) OR (intracranial hemorrhage[MeSH Terms]) OR (ICH[Title/Abstract]) OR (Intracranial hemorrhage[Title/Abstract]) OR (SAH[Title/Abstract]) OR (subarachnoid haemorrhage[Title/Abstract]) OR (intracerebral bleeding[Title/Abstract]) OR (microbleed*[Title/Abstract]) OR (micro bleed*[Title/Abstract])) AND ((sars virus[MeSH Terms]) OR (SARS*[Title/Abstract]) OR (COVID*[Title/Abstract]))
