# Supplementary material for: COVID-19 and Intracranial Hemorrhage: A Multicenter Case Series, Systematic Review and Pooled Analysis
Source: J Clin Med. 2022 Jan 25;11(3):605. doi: 10.3390/jcm11030605 (PMC8836638; doi:10.3390/jcm11030605)
Supplement: Supplementary file 1 [file jcm-11-00605-s001.zip › Supplementary File S2.pdf]

Supplemental 2

author	year of publication	events	incidence [%]	mortality [%]	age [years]	gender	stage of disease	fever	respiratory symptoms	malaise	myalgia/arthrarthralgia	focal deficits	altered mental state/level of	encephalopathy	anisocoria/abnormal pupils	headache	seizure	microbleeds	sah	iph	ivh	edh/sdh	ht/ph	svt	others/not specified	multilocular, not further	anticoaghulation	ecmo
Agrawal et al. [1]	2020	1	NK	NK	56	m	Uncomplicated	1	1	0	0	1	1	0	0	0	0	0	0	1	0	0	0	0	0	NK	0	0
Agrawal et al. [1]	2020	1	NK	NK	72	m	Uncomplicated	0	0	0	0	1	0	0	0	0	0	0	0	1	0	0	0	0	0	NK	0	0
Saieght et al. [2]	2020	1	NK	NK	31	m	Uncomplicated	1	1	1	1	0	1	0	0	1	0	0	1	0	0	0	0	0	0	NK	0	0
Saieght et al. [2]	2020	1	NK	NK	61	f	Uncomplicated	0	0	0	0	0	1	0	0	0	0	0	0	0	0	0	1	0	0	NK	0	0
Al Dalahmah et al. [3]	2020	1	NK	NK	73	m	Uncomplicated	0	0	0	0	0	1	0	0	1	0	0	0	0	0	0	0	0	0	NK	0	0
Al-olama et al. [4]	2020	1	NK	NK	36	m	Uncomplicated	1	1	0	1	0	0	1	0	1	0	0	0	0	0	1	0	0	0	NK	0	0
Benger et al. [5]	2020	1	NK	NK	41	m	Recovery	1	1	1	0	1	1	0	0	0	0	0	0	1	0	0	0	0	0	NK	pHEP	NK
Benger et al. [5]	2020	1	NK	NK	54	f	Uncomplicated	0	1	0	0	1	0	0	0	0	0	0	0	1	0	0	0	0	0	NK	tHEP	0
Benger et al. [5]	2020	1	NK	NK	50	m	Critical Phase	0	1	0	0	0	0	0	0	0	NK	0	0	1	0	0	0	0	0	NK	pHEP	NK
Benger et al. [5]	2020	1	NK	NK	64	f	Critical Phase	1	1	0	0	0	1	0	0	0	0	0	0	1	0	0	0	0	0	NK	1	1
Benger et al. [5]	2020	1	NK	NK	52	m	Critical Phase	1	1	0	0	0	1	0	0	0	0	0	0	1	0	0	0	0	0	NK	1	1
Cannac et al. [6]	2020	1	NK	NK	63	m	NK	NK	NK	NK	NK	NK	NK	NK	NK	NK	NK	1	0	0	0	0	0	0	0	NK	NK	1
Caroll et al. [7]	2020	1	NK	NK	62	m	Critical	1	1	0	0	0	1	0	0	0	0	0	0	1	0	0	0	0	0	NK	1	0
Caroll et al. [7]	2020	1	NK	NK	74	m	Critical	0	1	0	0	1	1	0	0	0	0	0	0	1	0	0	0	0	0	NK	1	0
Chougar et al. [8]	2020	1	NK	NK	72	m	Uncomplicated	0	1	0	0	1	1	0	0	0	1	0	0	0	0	0	0	1	0	NK	0	0
Daci et al. [9]	2020	1	NK	NK	60	f	Uncomplicated	0	1	1	0	0	1	0	0	1	0	0	0	1	0	0	0	0	0	NK	0	0

Dakay et al. [10]	2020	1	NK	NK	35	f	Uncomplicated	0	1	0	0	0	0	0	0	1	0	0	1	0	0	0	0	0	0	NK	0	0
De Stefano et al. [11]	2020	1	NK	NK	56	f	Critical	1	1	0	0	0	0	0	0	0	0	1	0	0	0	0	0	0	0	NK	NK	0
Dixon et al. [12]	2020	1	NK	NK	59	f	Uncomplicated	1	1	0	1	0	0	0	0	1	1	1	0	0	0	0	0	0	0	NK	0	0
Franceschi et al. [13]	2020	1	NK	NK	48	m	Critical	1	1	0	0	0	1	0	0	0	0	1	0	0	0	0	0	0	0	NK	NK	0
Franceschi et al. [13]	2020	1	NK	NK	67	f	Uncomplicated	NK	NK	NK	NK	0	1	0	0	0	0	1	0	0	0	0	0	0	0	NK	NK	0
Ghani et al. [14]	2020	1	NK	NK	59	m	Critical	0	1	0	0	1	0	0	0	0	0	0	1	1	0	0	0	0	0	NK	1	NK
Ghani et al. [14]	2020	1	NK	NK	61	f	Critical	0	1	0	0	1	0	0	0	0	0	0	0	0	0	1	0	0	0	NK	tHEP	NK
Ghani et al. [14]	2020	1	NK	NK	59	f	Critical	0	1	0	0	0	0	0	0	0	0	0	0	1	0	0	0	0	0	NK	tHEP	NK
Ghosh et al. [14]	2020	1	NK	NK	44	f	Uncomplicated	0	0	0	0	0	0	0	0	0	1	0	0	0	0	0	0	1	0	NK	0	0
Gogia et al. [15]	2020	1	NK	NK	75	f	Critical	0	1	0	0	0	0	0	0	0	NK	0	0	0	0	1	0	0	0	NK	tHEP	NK
Goncalves et al. [16]	2020	1	NK	NK	56	f	Critical	1	1	0	0	0	0	0	1	0	0	0	0	0	0	0	0	1	0	NK	pHEP	0
Goncalves et al. [16]	2020	1	NK	NK	40	m	Critical	0	1	0	0	0	0	0	1	0	0	0	0	1	0	0	0	0	0	NK	pHEP	0
Goncalves et al. [16]	2020	1	NK	NK	60	f	Critical	0	1	0	0	0	0	0	1	0	0	0	0	1	0	0	0	0	0	NK	1	0
Gupta et al. [17]	2020	1	NK	NK	45	m	Critical	1	1	0	0	0	0	1	0	0	0	1	0	0	0	0	0	0	0	NK	NK	1
Harrogate et al. [18]	2020	1	NK	NK	74	m	Critical	0	1	0	0	0	1	0	0	0	0	0	1	0	0	0	0	0	0	NK	pHEP	0
Harrogate et al. [18]	2020	1	NK	NK	53	m	Critical	0	1	0	0	0	0	0	0	0	1	0	1	0	0	0	0	0	0	NK	pHEP	0
Heman-Ackah et al. [19]	2020	1	NK	NK	58	f	Critical	1	1	0	0	0	0	0	1	0	0	0	0	1	0	0	0	0	0	NK	1	1
Heman-Ackah et al. [19]	2020	1	NK	NK	46	m	Critical	1	1	0	0	0	0	0	0	1	0	0	0	1	0	0	0	0	0	NK	1	1
Hemasian et al. [20]	2020	1	NK	NK	65	m	Uncomplicated	0	0	0	0	0	1	0	0	0	0	0	0	0	0	0	0	1	0	NK	0	0
Hernández-Fernández et al. [21]	2020	1	NK	NK	51	f	NK	NK	NK	NK	NK	1	1	NK	NK	NK	0	0	0	1	0	0	0	0	0	NK	0	NK
Hernández-Fernández et al. [21]	2020	1	NK	NK	69	m	Critical	1	1	0	0	0	1	0	0	0	0	0	0	1	0	0	0	0	0	NK	tHEP	NK
Hernández-Fernández et al. [21]	2020	1	NK	NK	61	m	Critical	0	1	0	0	0	0	0	0	0	1	0	1	1	0	0	0	0	0	NK	tHEP	NK
Hernández-Fernández et al. [21]	2020	1	NK	NK	64	m	Critical	0	1	0	0	0	1	0	0	0	0	0	1	1	0	0	0	0	0	NK	tHEP	NK
Hernández-Fernández et al. [21]	2020	1	NK	NK	68	m	Critical	0	1	0	0	0	1	0	0	0	0	0	1	1	0	0	0	0	0	NK	NK	NK
Hernández-Fernández et al. [21]	2020	1	NK	NK	NK	NK	Critical	0	1	0	0	0	1	0	0	0	1	1	0	0	0	0	0	0	0	NK	NK	NK
Khattar et al. [22]	2020	1	NK	NK	42	m	Critical	1	1	0	0	0	0	0	1	0	0	0	0	1	0	0	0	0	0	NK	1	0
Lévesque et al. [23]	2020	1	NK	NK	53	m	Critical	1	1	0	0	0	0	0	0	0	0	0	0	1	0	0	0	0	0	NK	1	0
Li et al. [24]	2020	1	NK	NK	68	m	Critical	1	1	1	0	0	1	0	0	0	0	0	0	1	0	0	0	0	0	NK	1	0

Montes-Ramirez et al. [25]	2020	1	NK	NK	27	f	Recovery	1	1	0	0	1	0	0	0	0	0	1	0	0	0	0	0	0	0	NK	1	0
Motoie et al. [26]	2020	1	NK	NK	55	m	Critical	1	1	0	1	0	0	0	1	0	0	0	0	1	0	0	0	0	0	NK	1	1
Muhammad et al. [27]	2020	1	NK	NK	60	f	Uncomplicated	0	0	0	0	0	1	0	0	0	0	0	1	1	0	0	0	0	0	NK	0	0
Planinc et al. [28]	2020	1	NK	NK	31	m	Critical	1	1	0	0	1	0	0	0	1	0	1	0	0	0	0	0	0	0	NK	pHEP	0
Shekhar et al. [29]	2020	1	NK	NK	38	f	Critical	1	1	0	0	0	1	0	0	0	0	0	1	0	0	0	0	0	0	NK	pHEP	NK
Shekhar et al. [29]	2020	1	NK	NK	47	f	Critical	1	1	0	0	0	1	0	1	0	0	0	1	0	0	0	1	0	0	NK	pHEP	NK
Shekhar et al. [29]	2020	1	NK	NK	58	f	Critical	1	1	0	0	0	1	0	1	0	0	0	1	1	0	0	0	0	0	NK	pHEP	NK
Shoskes et al. [29]	2020	1	NK	NK	69	m	Critical	1	1	0	0	0	1	0	0	0	0	1	0	0	0	0	0	0	0	NK	NK	0
Soldatelli et al. [30]	2020	1	NK	NK	67	m	Critical	1	1	0	0	0	1	0	0	0	0	1	1	1	0	0	0	0	0	NK	1	0
Thu et al. [31]	2020	1	NK	NK	72	m	Uncomplicated	0	0	0	0	0	0	0	0	0	1	0	0	1	0	0	0	0	0	NK	0	0
Usman et al. [32]	2020	1	NK	NK	54	f	Critical	0	1	0	0	0	0	0	1	0	0	0	1	1	0	0	0	0	0	NK	1	0
Usman et al. [32]	2020	1	NK	NK	54	m	Critical	0	1	0	0	0	0	0	1	0	0	0	0	1	0	0	0	0	0	NK	1	0
Usman et al. [32]	2020	1	NK	NK	54	m	Critical	0	1	0	0	1	0	0	1	0	0	0	0	1	0	0	0	0	0	NK	1	0
Usman et al. [32]	2020	1	NK	NK	54	m	Critical	0	1	0	0	0	1	0	0	0	0	0	1	0	0	0	0	0	0	NK	1	0
Vattoth et al. [33]	2020	1	NK	NK	66	f	Critical	1	1	1	0	0	1	0	0	0	0	1	0	0	0	0	0	0	0	NK	NK	0
Vu et al. [34]	2020	1	NK	NK	30	m	Uncomplicated	0	0	0	0	1	0	0	0	0	0	0	0	1	0	0	0	0	0	NK	0	0
Wee et al. [35]	2020	1	NK	NK	64	m	Critical	0	1	0	0	0	0	0	1	0	0	0	0	1	0	0	0	0	0	NK	1	1
Zulfiqar et al. [36]	2020	1	NK	NK	65	f	Complicated	1	1	1	0	0	0	0	0	1	0	0	1	0	0	0	0	0	0	NK	pHEP	0
Sharifi-Razavi et al. [37]	2020	1	NK	NK	79	m	Complicated	1	1	0	0	0	1	0	0	0	0	0	1	1	0	0	0	0	0	NK	0	0
Rustemi et al. [38]	2020	1	NK	NK	68	f	Uncomplicated	1	0	0	0	0	0	0	0	1	0	0	1	0	0	0	0	0	0	NK	0	0
Batcik et al. [39]	2021	1	NK	NK	52	f	Critical	1	0	0	0	0	1	0	0	0	0	0	1	0	0	0	0	0	0	NK	0	0
Batcik et al. [39]	2021	1	NK	NK	54	m	Critical	1	0	0	0	0	1	0	1	0	0	0	1	0	0	0	0	0	0	NK	0	0
Batcik et al. [39]	2021	1	NK	NK	36	f	Uncomplicated	0	0	0	0	0	1	0	0	0	0	0	1	0	0	0	0	0	0	NK	0	0
Cezar-Junior et al. [40]	2020	1	NK	NK	36	f	Uncomplicated	1	1	0	0	0	0	0	0	1	0	0	1	0	0	0	0	0	0	NK	0	0
Cezar-Junior et al. [40]	2020	1	NK	NK	53	m	Complicated	1	1	0	0	0	0	0	0	1	0	0	1	0	0	0	0	0	0	NK	0	0
Cezar-Junior et al. [40]	2020	1	NK	NK	61	m	Complicated	1	1	0	1	0	0	0	0	0	NK	0	1	0	0	0	0	0	0	NK	0	0
Cezar-Junior et al. [40]	2020	1	NK	NK	71	f	Complicated	1	NK	0	1	1	0	0	0	1	0	0	1	0	0	0	0	0	0	NK	0	0
Fayed et al. [41]	2020	1	NK	NK	57	f	Critical	1	1	0	0	0	0	1	0	0	0	0	0	1	0	0	0	0	0	NK	1	0

Fayed et al. [41]	2020	1	NK	NK	54	f	Critical	1	NK	0	1	0	0	0	1	0	0	0	1	0	0	0	0	0	0	NK	1	0
Fayed et al. [41]	2020	1	NK	NK	71	m	Critical	0	1	0	0	0	0	1	0	NK	0	0	0	1	0	0	0	0	0	NK	NK	NK
Gosh et al. [42]	2020	1	NK	NK	19	f	Uncomplicated	1	NK	1	0	1	1	0	0	1	0	0	0	1	0	0	0	0	0	NK	0	0
Haroon et al. [43]	2020	1	NK	NK	71	m	Critical	1	1	0	0	0	1	0	0	0	0	1	0	0	0	0	0	0	0	NK	1	0
Mousa-Ibrahim et al.[44]	2020	1	NK	NK	54	f	Critical	0	1	0	0	0	0	0	0	0	0	0	0	1	0	0	0	0	0	NK	1	0
Mousa-Ibrahim et al. [44]	2020	1	NK	NK	68	f	Critical	NK	NK	NK	NK	1	0	0	0	0	0	0	0	0	0	1	0	0	0	NK	1	0
Mousa-Ibrahim et al. [44]	2020	1	NK	NK	76	m	Critical	1	1	0	0	0	0	0	1	0	0	0	0	1	0	0	0	0	0	NK	1	0
Mousa-Ibrahim et al. [44]	2020	1	NK	NK	71	f	Critical	1	1	0	0	0	1	0	1	1	0	0	0	1	0	0	0	0	0	NK	1	0
Mousa-Ibrahim et al. [44]	2020	1	NK	NK	79	m	Critical	1	1	0	0	0	0	0	0	0	1	0	1	0	0	0	0	0	0	NK	tHEP	0
Mousa-Ibrahim et al. [44]	2020	1	NK	NK	63	m	Critical	1	1	0	0	0	0	0	0	1	0	0	0	1	0	0	0	0	0	NK	1	0
Nicolas-Jilwan et al. [45]	2020	1	NK	NK	59	m	Critical	0	1	0	0	NK	NK	NK	NK	NK	0	1	0	0	0	0	0	0	0	NK	NK	NK
Ordinola et al. [46]	2020	1	NK	NK	88	f	Uncomplicated	0	0	0	0	1	0	0	0	0	0	0	0	1	0	0	0	0	0	NK	pHEP	NK
Pavlov et al. [47]	2020	1	NK	NK	56	m	Critical	0	1	1	0	1	1	0	0	1	0	0	0	1	0	0	0	0	0	NK	pHEP	NK
Pavlov et al. [47]	2020	1	NK	NK	64	m	Critical	0	1	1	0	1	0	0	0	0	0	0	0	1	0	0	0	0	0	NK	NK	NK
Pavlov et al. [47]	2020	1	NK	NK	60	m	Critical	1	1	0	0	0	1	0	0	1	0	0	0	1	0	0	0	0	0	NK	NK	NK
Tristán-Samaniego et al. [48]	2020	1	NK	NK	46	m	Critical	1	1	0	0	0	1	0	0	0	0	1	0	0	0	0	0	0	0	NK	NK	NK
Daneshi et al. [49]	2020	1	NK	NK	34	f	Uncomplicated	NK	NK	Nk	Nk	Nk	1	Nk	Nk	Nk	0	0	0	0	1	0	0	0	0	NK	NK	NK
Daneshi et al. [49]	2020	1	NK	NK	60	m	Uncomplicated	NK	NK	Nk	Nk	Nk	Nk	Nk	Nk	1	0	0	0	1	1	0	0	0	0	NK	NK	NK
Daneshi et al. [49]	2020	1	NK	NK	63	f	Uncomplicated	NK	NK	Nk	Nk	Nk	1	Nk	Nk	Nk	0	0	0	1	1	0	0	0	0	NK	NK	NK
Daneshi et al. [49]	2020	1	NK	NK	85	f	NK	Nk	Nk	Nk	Nk	1	0	Nk	Nk	Nk	0	0	0	0	0	0	1	0	0	NK	NK	NK
Daneshi et al. [49]	2020	1	NK	NK	56	m	Critical	1	1	0	0	0	1	0	0	0	0	NK	1	0	0	0	0	0	0	NK	tHEP	1
Kirschenbaum et al. [50]	2020	1	NK	NK	70	m	Critical	NK	NK	NK	NK	NK	1	NK	1	NK	0	1	0	0	0	0	0	0	0	NK	1	NK
Kirschenbaum et al. [50]	2020	1	NK	NK	77	f	Critical	NK	NK	NK	NK	NK	0	0	0	0	0	1	0	0	0	0	0	0	0	NK	pHEP	NK
Kirschenbaum et al. [50]	2020	1	NK	NK	79	m	Critical	NK	NK	NK	NK	NK	0	0	0	0	0	1	0	0	0	0	0	0	0	NK	tHEP	NK
Kirschenbaum et al. [50]	2020	1	NK	NK	81	m	Critical	NK	NK	NK	NK	NK	1	0	0	0	0	1	0	0	0	0	0	0	0	NK	pHEP	NK
Mishra et al. [51]	2020	1	NK	NK	30	m	NK	NK	NK	NK	NK	NK	NK	NK	NK	NK	NK	NK	0	0	0	0	0	0	0	NK	NK	NK
Mishra et al. [51]	2020	1	NK	NK	89	m	NK	NK	NK	NK	NK	NK	NK	NK	NK	NK	NK	NK	0	0	0	0	0	0	0	NK	NK	NK

Mishra et al. [51]	2020	1	NK	NK	66	m	NK	NK	NK	NK	NK	NK	NK	NK	NK	NK	NK	NK	0	0	0	0	0	0	0	NK	NK	NK
Mishra et al. [51]	2020	1	NK	NK	89	m	NK	NK	NK	NK	NK	NK	NK	NK	NK	NK	NK	NK	0	0	0	0	0	0	0	NK	NK	NK
Mishra et al. [51]	2020	1	NK	NK	69	f	NK	NK	NK	NK	NK	NK	NK	NK	NK	NK	NK	NK	0	0	0	0	0	0	0	NK	NK	NK
Mishra et al. [51]	2020	1	NK	NK	83	m	NK	NK	NK	NK	NK	NK	NK	NK	NK	NK	NK	NK	0	0	0	0	0	0	0	NK	NK	NK
Mishra et al. [51]	2020	1	NK	NK	70	m	NK	NK	NK	NK	NK	NK	NK	NK	NK	NK	NK	NK	0	0	0	0	0	0	0	NK	NK	NK
Mishra et al. [51]	2020	1	NK	NK	72	f	NK	NK	NK	NK	NK	NK	NK	NK	NK	NK	NK	NK	0	0	0	0	0	0	0	NK	NK	NK
Mishra et al. [51]	2020	1	NK	NK	95	f	NK	NK	NK	NK	NK	NK	NK	NK	NK	NK	NK	NK	0	0	0	0	0	0	0	NK	NK	NK
Mishra et al. [51]	2020	1	NK	NK	32	m	NK	NK	NK	NK	NK	NK	NK	NK	NK	NK	NK	NK	0	0	0	0	0	0	0	NK	NK	NK
Mishra et al. [51]	2020	1	NK	NK	79	f	NK	NK	NK	NK	NK	NK	NK	NK	NK	NK	NK	NK	0	0	0	0	0	0	0	NK	NK	NK
Agrawal et al. [52]	2020	25	NK	NK	NK	NK	NK	NK	NK	NK	NK	NK	NK	NK	NK	NK	NK	25	NK	NK	NK	NK	NK	NK	NK	NK	NK	NK
Altschul et al. [53]	2020	35	NK	45.70%	67	NK	NK	6	11	NK	NK	NK	NK	NK	NK	NK	NK	NK	2	9	NK	17	NK	NK	NK	7	7	NK
Coolen et al. [54]	2020	19	NK	NK	77	NK	NK	5	18	NK	NK	NK	NK	5	NK	2	1	1	NK	1	NK	NK	NK	NK	NK	NK	NK	NK
D'Amore et al. [55]	2020	9	NK	NK	NK	NK	NK	NK	NK	NK	NK	NK	NK	NK	NK	NK	NK	0	1	2	0	2	2	0	2	NK	0	NK
Dogra et al. [56]	2020	33	4.37%	42.42%	62	NK	NK	NK	NK	NK	NK	12	NK	17	NK	NK	2	7	NK	26	NK	NK	NK	NK	NK	NK	32	NK
Fitisiori et al. [57]	2020	9	NK	NK	68	NK	NK	8	6	NK	NK	NK	3	6	NK	NK	NK	9	NK	NK	NK	NK	NK	NK	NK	NK	NK	NK
Katz et al. [58]	2020	29	NK	0.27%	NK	NK	NK	NK	NK	NK	NK	NK	NK	NK	NK	NK	NK	NK	NK	NK	NK	NK	5	1	23	NK	NK	NK
Kirschenbaum et al. [50]	2020	6	NK	NK	NK	NK	Critical	NK	NK	NK	NK	2	4	NK	NK	NK	NK	3	3	NK	NK	NK	NK	NK	NK	NK	NK	NK
Lin et al. [59]	2020	41	2.00%	NK	NK	NK	NK	NK	NK	NK	NK	NK	NK	NK	NK	NK	NK	26	2	10	NK	3	NK	NK	NK	NK	NK	NK
Masur et al. [60]	2020	5	NK	100.00 %	NK	NK	Critical	NK	NK	NK	NK	NK	NK	NK	NK	NK	NK	NK	5	NK	NK	NK	NK	NK	NK	5	NK	NK
Nawabi et al. [61]	2020	18	NK	NK	49.5 0	NK	NK	NK	NK	NK	NK	NK	NK	NK	NK	NK	NK	NK	9	6	2	1	NK	NK	NK	NK	NK	NK
Radmanesh et al. [62]	2020	7	25.93 %	NK	NK	NK	Critical	NK	NK	NK	NK	NK	NK	NK	NK	NK	NK	7	NK	NK	NK	NK	NK	NK	NK	NK	NK	NK
Requena et al. [63]	2020	4	0.20%	NK	NK	NK	NK	NK	NK	NK	NK	NK	NK	NK	NK	NK	NK	NK	NK	NK	NK	NK	NK	NK	NK	NK	NK	NK
Rothstein et al. [64]	2020	8	0.95%	75.00%	NK	NK	NK	NK	NK	NK	NK	NK	NK	NK	NK	NK	NK	NK	3	5	NK	NK	NK	NK	NK	NK	NK	4
Sawlani et al. [65]	2020	18	0.53%	NK	NK	NK	NK	NK	NK	NK	NK	NK	NK	NK	NK	NK	NK	12	1	2	NK	NK	NK	NK	3	NK	NK	NK
Siegler et al. [66]	2020	42	0.22%	33.33%	NK	NK	NK	NK	NK	NK	NK	NK	NK	NK	NK	NK	NK	NK	NK	NK	NK	NK	NK	NK	NK	NK	NK	NK
Trifan et al. [67]	2020	19	NK	50.00%	NK	NK	NK	NK	NK	NK	NK	NK	NK	NK	NK	NK	NK	NK	3	NK	NK	NK	NK	NK	16	NK	NK	NK

Sweid et al. [68]	2020	3	NK	NK	NK	NK	NK	NK	NK	NK	NK	NK	NK	NK	NK	NK	NK	NK	NK	NK	NK	NK	NK	NK	NK	NK	NK	NK
Bermea et al. [69]	2021	11	NK	81.82%	53	NK	Critical	NK	NK	NK	NK	NK	NK	NK	NK	NK	NK	NK	NK	NK	NK	NK	NK	NK	11	NK	11	11
Büttner et al. [70]	2021	9	1.59%	NK	NK	NK	NK	NK	NK	NK	NK	NK	NK	NK	NK	NK	NK	7	4	2	NK	NK	NK	NK	5	NK	NK	NK
Dixon et al. [71]	2020	10	NK	NK	56	NK	NK	NK	NK	NK	NK	3	6	NK	NK	NK	1	10	NK	NK	NK	NK	NK	NK	NK	NK	NK	1
John et al. [72]	2020	12	NK	16.67%	48.1	NK	NK	NK	NK	NK	NK	NK	NK	NK	NK	NK	NK	NK	NK	NK	NK	NK	NK	NK	NK	NK	NK	NK
Lang et al. [73]	2020	9	NK	NK	NK	NK	Critical	NK	NK	NK	NK	NK	NK	NK	NK	NK	NK	NK	2	2	NK	1	NK	NK	NK	4	NK	NK
Lawton et al. [74]	2021	22	NK	45.45%	54.27	NK	NK	NK	NK	NK	NK	NK	NK	NK	NK	NK	NK	NK	NK	NK	NK	NK	NK	NK	NK	NK	NK	NK
Lersy et al. [75]	2020	19	NK	21.05%	66	NK	NK	NK	NK	NK	NK	3	17	NK	NK	NK	4	NK	NK	NK	NK	NK	NK	NK	NK	NK	NK	NK
Shahjouei et al. [76]	2020	27	0.10%	NK	63	NK	NK	NK	NK	NK	NK	NK	NK	NK	NK	NK	NK	NK	NK	NK	NK	NK	NK	NK	NK	NK	NK	NK
PANDEMIC_001	2021	1	NK	NK	46	m	Critical	0	0	0	0	1	1	0	1	0	0	0	1	1	0	0	0	0	0	0	tHEP	1
PANDEMIC_002	2021	1	NK	NK	61	m	Critical	0	0	0	0	0	1	0	0	0	0	0	1	0	0	0	0	0	0	0	tHEP	1
PANDEMIC_003	2021	1	NK	NK	66	m	Complicated	0	0	0	0	0	1	0	1	0	0	0	0	1	0	0	0	0	0	0	tHEP	1
PANDEMIC_004	2021	1	NK	NK	71	f	Critical	0	0	0	0	0	1	0	1	0	0	0	1	1	0	0	0	0	0	0	pHEP	1
PANDEMIC_005	2021	1	NK	NK	80	m	Critical	0	0	0	0	0	1	0	1	0	0	0	1	1	0	0	0	0	0	0	tHEP	0
PANDEMIC_006	2021	1	NK	NK	64	m	Critical	0	0	0	0	0	1	1	0	0	0	1	1	1	0	0	0	0	0	0	tHEP	1
PANDEMIC_007	2021	1	NK	NK	86	m	Critical	0	1	0	0	1	0	0	0	0	0	0	0	1	0	0	0	0	0	0	tHEP	0
PANDEMIC_008	2021	1	NK	NK	85	f	Uncomplicated	0	0	0	0	1	0	0	0	0	0	0	1	1	0	0	0	0	0	0	0	0
PANDEMIC_009	2021	1	NK	NK	70	m	Complicated	1	0	0	0	1	1	0	1	0	0	0	0	0	0	1	0	0	0	0	pHEP	0
PANDEMIC_010	2021	1	NK	NK	76	f	Complicated	0	1	0	0	1	0	0	0	0	0	0	0	1	0	0	0	0	0	0	pHEP	0
PANDEMIC_011	2021	1	NK	NK	76	m	Critical	1	1	0	0	0	1	0	0	0	0	0	0	1	0	0	0	0	0	0	pHEP	0
PANDEMIC_012	2021	1	NK	NK	54	m	Critical	0	1	0	0	0	0	0	1	0	0	0	1	1	0	0	0	0	0	0	tHEP	0
PANDEMIC_013	2021	1	NK	NK	68	m	Critical	0	1	0	0	0	0	0	0	0	0	0	1	0	0	1	0	0	0	0	tHEP	0
PANDEMIC_014	2021	1	NK	NK	72	f	Critical	0	1	0	0	1	0	0	0	0	0	0	1	1	1	0	0	0	0	0	tHEP	1
PANDEMIC_015	2021	1	NK	NK	57	m	Recovery	0	1	0	0	0	1	0	0	0	0	0	0	0	1	0	0	0	0	0	tHEP	0
PANDEMIC_016	2021	1	NK	NK	60	m	Critical	0	1	0	0	0	1	0	0	0	0	0	1	0	0	0	0	0	0	0	0	0
PANDEMIC_017	2021	1	NK	NK	57	m	Critical	0	1	0	0	0	1	0	1	0	0	0	1	0	0	0	0	0	0	0	tHEP	1

PANDEMIC_018	2021	1	NK	NK	78	m	Critical	0	1	0	0	0	0	0	0	0	0	0	0	1	0	0	0	0	0	0	1	0
PANDEMIC_019	2021	1	NK	NK	49	m	Critical	0	1	0	0	0	1	0	1	0	0	0	0	1	0	1	0	0	0	0	tHEP	1
PANDEMIC_020	2021	1	NK	NK	82	m	Uncomplicated	0	1	0	0	0	1	0	0	0	0	1	0	0	0	0	0	0	0	0	1	0
PANDEMIC_021	2021	1	NK	NK	78	m	Uncomplicated	0	1	0	0	0	1	0	0	0	0	1	0	0	0	0	0	0	0	0	1	0
PANDEMIC_022	2021	1	NK	NK	51	f	Critical	0	1	0	0	0	0	0	0	0	0	0	0	1	0	0	0	0	0	0	1	1
PANDEMIC_023	2021	1	NK	NK	59	m	Uncomplicated	0	0	1	1	0	0	0	0	0	0	1	0	0	0	0	0	0	0	0	1	0
PANDEMIC_024	2021	1	NK	NK	60	m	Uncomplicated	0	0	1	1	0	1	0	0	0	0	0	0	1	0	0	0	0	0	0	1	0
PANDEMIC_025	2021	1	NK	NK	78	m	Uncomplicated	0	0	1	1	0	1	0	0	0	0	0	0	0	1	0	0	0	0	0	1	0
PANDEMIC_026	2021	1	NK	NK	56	m	Complicated	0	1	0	0	0	1	0	0	0	0	1	0	0	0	0	0	0	0	0	1	1
PANDEMIC_027	2021	1	NK	NK	40	m	Complicated	0	1	0	0	0	1	0	0	0	0	0	1	0	0	0	0	0	0	0	1	0
PANDEMIC_028	2021	1	NK	NK	53	m	Critical	0	1	0	0	0	0	0	0	0	0	0	0	1	0	0	0	0	0	0	1	1
PANDEMIC_029	2021	1	NK	NK	64	m	Critical	1	1	0	0	0	1	0	1	0	0	0	0	1	0	0	0	0	0	0	tHEP	0
PANDEMIC_030	2021	1	NK	NK	66	m	Critical	0	1	0	0	0	0	0	0	0	0	1	1	0	0	0	0	0	0	0	1	0
PANDEMIC_031	2021	1	NK	NK	85	m	Critical	0	1	0	0	0	1	0	0	0	0	0	1	0	0	1	0	0	0	0	pHEP	0
PANDEMIC_032	2021	1	NK	NK	42	m	Critical	0	0	0	0	0	0	0	1	0	0	0	1	1	0	0	0	0	0	0	tHEP	1
PANDEMIC_033	2021	1	NK	NK	63	m	Critical	0	0	0	0	0	0	0	1	0	0	0	1	1	0	0	0	0	0	0	tHEP	0
PANDEMIC_034	2021	1	NK	NK	62	m	Critical	0	0	0	0	0	0	0	1	0	0	0	0	1	1	0	0	0	0	0	tHEP	0

NK not known; SAH subarachnoid hemorrhage; IPH intraparenchymal hemorrhage; IVH intraventricular hemorrhage; EDH/SDH epidural/subdural hematoma; HT/PH hemorrhagic transformation/parenchymal hematoma in ischemic stroke; SVT cerebral sinus thrombosis; ECMO extracorporeal membrane oxygenation; tHEP therapeutic range heparinization/therapeutic range anticoagulation; pHEP prophylactic range heparinization;

author	year of publication	crp [mg/L]	leukocytes [109/L]	inr	aptt [seconds]	thrombocytes [109/L]	d-dimer [mg/L]	imaging	ICH diagnosis after COVID-19 diagnosis [days]	mrs
Agrawal et al. [1]	2020	41	NK	1	NK	220	NK	CT	-1	NK
Agrawal et al. [1]	2020	NK	NK	1	NK	165	NK	CT/MRI	NK	6
Saieght et al. [2]	2020	NK	NK	NK	NK	NK	NK	CT	7	NK
Saieght et al. [2]	2020	NK	NK	NK	NK	NK	NK	CT	NK	NK
Al Dalahmah et al. [3]	2020	9	13	NK	NK	346	NK	CT	NK	6
Al-olama et al. [4]	2020	NK	13	1	30	NK	1	CT	6	NK
Benger et al. [5]	2020	100	20	1	NK	510	2	CT	37	6
Benger et al. [5]	2020	7	6	3	NK	270	1	CT	14	5
Benger et al. [5]	2020	138	15	1	NK	72	9	CT	32	5
Benger et al. [5]	2020	330	16	1	NK	221	8	CT	32	5
Benger et al. [5]	2020	77	15	1	NK	313	8	CT	38	5
Cannac et al. [6]	2020	NK	NK	NK	NK	NK	NK	MRI	NK	NK
Caroll et al. [7]	2020	370	17	NK	NK	123	10	CT	20	6
Caroll et al. [7]	2020	415	18	NK	NK	210	5	CT	17	6
Chougar et al. [8]	2020	NK	NK	NK	NK	NK	NK	CT/MRI	3	6
Daci et al. [9]	2020	NK	NK	NK	NK	NK	NK	CT/MRI	NK	6
Dakay et al. [10]	2020	NK	NK	NK	NK	NK	NK	CT	12	0
De Stefano et al. [11]	2020	52	20	NK	NK	602	2	MRI	24	NK

Dixon et al. [12]	2020	144	2	NK	NK	29	NK	CT/MRI	10	6
Franceschi et al. [13]	2020	NK	NK	NK	NK	NK	NK	CT/MRI	18	NK
Franceschi et al. [13]	2020	NK	NK	NK	NK	NK	NK	CT/MRI	2	1
Ghani et al. [14]	2020	NK	NK	NK	57	NK	>3	CT	18	6
Ghani et al. [14]	2020	NK	NK	NK	63	NK	NK	CT	10	6
Ghani et al. [14]	2020	NK	NK	NK	NK	NK	NK	CT	15	6
Ghosh et al. [14]	2020	NK	NK	NK	NK	NK	NK	CT/MRI	NK	6
Gogia et al. [15]	2020	352	10	1	22	315	1	CT	NK	6
Goncalves et al. [16]	2020	NK	NK	NK	59	NK	6	CT	15	6
Goncalves et al. [16]	2020	NK	NK	NK	NK	NK	6	CT	16	6
Goncalves et al. [16]	2020	NK	NK	2	NK	NK	NK	CT	15	6
Gupta et al. [17]	2020	47	NK	NK	NK	NK	0	MRI	23	6
Harrogate et al. [18]	2020	NK	NK	NK	NK	36	55	CT/MRI	11	6
Harrogate et al. [18]	2020	NK	NK	NK	NK	NK	19	CT	NK	6
Heman-Ackah et al. [19]	2020	NK	16	2	60	139	20	CT	19	6
Heman-Ackah et al. [19]	2020	NK	41	1	71	197	1	CT	16	6
Hemasian et al. [20]	2020	NK	NK	NK	NK	NK	NK	CT	1	6
Hernández-Fernández et al. [21]	2020	NK	NK	NK	NK	NK	NK	CT	1	6
Hernández-Fernández et al. [21]	2020	NK	NK	NK	NK	NK	NK	CT/MRI	13	NK
Hernández-Fernández et al. [21]	2020	NK	NK	NK	NK	NK	NK	CT	1	6
Hernández-Fernández et al. [21]	2020	NK	NK	NK	NK	NK	NK	CT/MRI	15	NK
Hernández-Fernández et al. [21]	2020	NK	NK	NK	NK	NK	NK	CT	16	NK
Hernández-Fernández et al. [21]	2020	NK	NK	NK	NK	NK	NK	CT/MRI	NK	6
Khattar et al. [22]	2020	310	NK	NK	110	120	Normal	CT	24	6
Lévesque et al. [23]	2020	NK	10	NK	NK	23	Normal	CT	39	6
Li et al. [24]	2020	NK	NK	2	NK	NK	11	CT	19	6
Montes-Ramirez et al. [25]	2020	NK	NK	NK	NK	NK	12	CT/MRI	15	0
Motoie et al. [26]	2020	NK	NK	NK	35	66	3	CT	5	6

Muhammad et al. [27]	2020	11	14	NK	NK	NK	Normal	CT	1	6
Planinc et al. [28]	2020	449	30	NK	NK	92	59	CT/MRI	13	3
Shekhar et al. [29]	2020	178	11	1	NK	263	0	CT	16	6
Shekhar et al. [29]	2020	54	11	1	NK	302	1	CT/MRI	6	6
Shekhar et al. [29]	2020	NK	16	1	NK	239	1	CT	19	6
Shoskes et al. [29]	2020	51	NK	NK	NK	NK	11	MRI	NK	6
Soldatelli et al. [30]	2020	NK	NK	NK	NK	NK	NK	MRI	NK	6
Thu et al. [31]	2020	164	NK	NK	NK	NK	NK	CT/MRI	1	0
Usman et al. [32]	2020	NK	NK	NK	60	NK	20	CT	22	6
Usman et al. [32]	2020	NK	NK	NK	71	NK	1	CT	7	6
Usman et al. [32]	2020	NK	NK	NK	38	NK	5	CT	3	6
Usman et al. [32]	2020	NK	NK	NK	61	NK	9	CT	4	6
Vattoth et al. [33]	2020	68	15	1	64	145	3	CT/MRI	NK	NK
Vu et al. [34]	2020	NK	NK	NK	NK	NK	NK	CT	1	6
Wee et al. [35]	2020	NK	NK	NK	NK	NK	NK	CT	NK	NK
Zulfiqar et al. [36]	2020	55	NK	NK	NK	2	NK	CT	9	0
Sharifi-Razavi et al. [37]	2020	10	NK	1	64	210	NK	CT	3	NK
Rustemi et al. [38]	2020	NK	NK	NK	NK	NK	NK	CT	1	0
Batcik et al. [39]	2021	NK	NK	NK	NK	NK	NK	CT	NK	6
Batcik et al. [39]	2021	NK	NK	NK	NK	NK	NK	CT	NK	6
Batcik et al. [39]	2021	NK	NK	NK	NK	NK	NK	CT	NK	6
Cezar-Junior et al. [40]	2020	40	NK	NK	NK	NK	4	CT	NK	4
Cezar-Junior et al. [40]	2020	34	NK	NK	NK	NK	1	CT	4	2
Cezar-Junior et al. [40]	2020	56	NK	NK	NK	NK	3	CT	23	6
Cezar-Junior et al. [40]	2020	27	NK	NK	NK	NK	1	CT	7	4
Fayed et al. [41]	2020	NK	NK	NK	NK	NK	NK	CT	14	6
Fayed et al. [41]	2020	NK	NK	NK	NK	NK	NK	CT	11	6
Fayed et al. [41]	2020	NK	NK	NK	NK	NK	3	CT	19	6

Gosh et al. [42]	2020	NK	NK	NK	NK	NK	NK	MRI	NK	6
Haroon et al. [43]	2020	23	18	NK	NK	NK	2	MRI	NK	5
Mousa-Ibrahim et al.[44]	2020	1070	10	1	46	131	20	CT	21	6
Mousa-Ibrahim et al. [44]	2020	NK	6	1	29	232	NK	CT	NK	6
Mousa-Ibrahim et al. [44]	2020	230	9	1	27	191	2	CT	7	6
Mousa-Ibrahim et al. [44]	2020	189	9	1	41	426	6	CT	26	6
Mousa-Ibrahim et al. [44]	2020	230	8	1	28	201	2	CT	10	6
Mousa-Ibrahim et al. [44]	2020	259	6	NK	NK	428	2	CT	8	6
Nicolas-Jilwan et al. [45]	2020	NK	NK	NK	NK	NK	NK	MRI	NK	6
Ordinola et al. [46]	2020	30	NK	NK	NK	NK	1	CT	4	5
Pavlov et al. [47]	2020	88	15	NK	NK	220	2	CT	21	4
Pavlov et al. [47]	2020	120	15	NK	NK	334	3	CT	10	2
Pavlov et al. [47]	2020	189	15	NK	NK	480	4	CT	12	NK
Tristán-Samaniego et al. [48]	2020	328	NK	NK	NK	NK	8	MRI	13	2
Daneshi et al. [49]	2020	NK	NK	NK	NK	NK	NK	NK	NK	6
Daneshi et al. [49]	2020	NK	NK	NK	NK	NK	NK	NK	NK	NK
Daneshi et al. [49]	2020	NK	NK	NK	NK	NK	NK	NK	NK	NK
Daneshi et al. [49]	2020	NK	NK	NK	NK	NK	NK	NK	NK	6
Daneshi et al. [49]	2020	220	Nk	NK	NK	NK	1.2	CT	NK	NK
Kirschenbaum et al. [50]	2020	299	NK	NK	NK	NK	2	Pathology	NK	6
Kirschenbaum et al. [50]	2020	145	NK	NK	NK	NK	NK	Pathology	NK	6
Kirschenbaum et al. [50]	2020	246	NK	NK	NK	238	NK	Pathology	NK	6
Kirschenbaum et al. [50]	2020	227	NK	NK	NK	NK	11	Pathology	NK	6
Mishra et al. [51]	2020	18	4.6	1	26.6	147	NK	CT/MRI	NK	NK
Mishra et al. [51]	2020	18	4.9	1	30.1	147	0.351	CT	NK	NK
Mishra et al. [51]	2020	149	27.1	1.6	24.7	380	NK	CT/MRI	NK	NK
Mishra et al. [51]	2020	55	5.1	1.1	26.1	148	NK	CT	NK	NK

Mishra et al. [51]	2020	18	20.9	1.2	22.3	192	58.804	CT	NK	6
Mishra et al. [51]	2020	41	11	2	29	275	0	CT	NK	6
Mishra et al. [51]	2020	88	25	3	66	274	3	CT	NK	6
Mishra et al. [51]	2020	19	16	1	71	344	3	CT/MRI	NK	NK
Mishra et al. [51]	2020	NK	6	NK	NK	89	NK	CT	NK	6
Mishra et al. [51]	2020	1	16	1	NK	230	NK	CT	NK	6
Mishra et al. [51]	2020	318	24	1	15	214	15	CT/MRI	NK	6
Agrawal et al. [52]	2020	NK	NK	NK	NK	NK	NK	NK	NK	NK
Altschul et al. [53]	2020	NK	NK	NK	NK	NK	NK	NK	NK	NK
Coolen et al. [54]	2020	NK	NK	NK	NK	NK	NK	MRI	NK	NK
D’Amore et al. [55]	2020	NK	NK	NK	NK	NK	NK	NK	NK	NK
Dogra et al. [56]	2020	NK	NK	1.3	77.0	200	2.204	NK	17	NK
Fitisiori et al. [57]	2020	NK	NK	NK	NK	NK	NK	MRT	27	NK
Katz et al. [58]	2020	NK	NK	NK	NK	NK	NK	CT/MRI	NK	NK
Kirschenbaum et al. [50]	2020	NK	NK	NK	NK	NK	NK	CT/MRI	NK	NK
Lin et al. [59]	2020	NK	NK	NK	NK	NK	NK	CT/MRI	NK	NK
Masur et al. [60]	2020	NK	NK	NK	NK	NK	NK	NK	11	NK
Nawabi et al. [61]	2020	240	9	NK	NK	218	9	CT	NK	NK
Radmanesh et al. [62]	2020	NK	NK	NK	NK	NK	NK	MRI	NK	NK
Requena et al. [63]	2020	NK	NK	NK	NK	NK	NK	NK	NK	NK
Rothstein et al. [64]	2020	NK	NK	NK	NK	NK	NK	NK	25	NK
Sawlani et al. [65]	2020	NK	NK	NK	NK	NK	NK	CT/MRI	NK	NK
Siegler et al. [66]	2020	NK	NK	NK	NK	NK	NK	CT/MRI	NK	NK
Trifan et al. [67]	2020	NK	NK	NK	NK	NK	NK	NK	NK	NK
Sweid et al. [68]	2020	NK	NK	NK	NK	NK	NK	NK	NK	NK
Bermea et al. [69]	2021	226	10.2	1.2	NK	275	5009	NK	NK	NK

Büttner et al. [70]	2021	NK	NK	NK	NK	NK	NK	NK	NK	NK
Dixon et al. [71]	2020	NK	NK	NK	NK	NK	NK	NK	37.5	NK
John et al. [72]	2020	NK	NK	1.1	29.2	220	NK	NK	NK	NK
Lang et al. [73]	2020	NK	NK	NK	NK	NK	NK	NK	NK	NK
Lawton et al. [74]	2021	NK	8590	NK	31.45	261363	NK	NK	NK	NK
Lersy et al. [75]	2020	NK	NK	NK	NK	NK	NK	NK	NK	NK
Shahjouei et al. [76]	2020									
PANDEMIC_001	2021	336	22	2	79	49	24	CT	43	6
PANDEMIC_002	2021	326	30	3	75	66	123	CT	14	NK
PANDEMIC_003	2021	331	25	1	126	261	6	CT	16	6
PANDEMIC_004	2021	499	8	3	60	31	35	CT	8	6
PANDEMIC_005	2021	208	15	1	30	214	35	CT	16	6
PANDEMIC_006	2021	232	31	1	77	136	35	MRI	79	4
PANDEMIC_007	2021	23	11	1	34	306	NK	CT	NK	5
PANDEMIC_008	2021	103	13	1	26	188	5	MRI	NK	NK
PANDEMIC_009	2021	151	20	2	39	186	NK	CT	6	6
PANDEMIC_010	2021	41	18	1	35	532	3	CT	23	6
PANDEMIC_011	2021	568	13	1	143	129	18	CT	35	5
PANDEMIC_012	2021	458	30	1	62	155	NK	CT	18	6
PANDEMIC_013	2021	340	21	2	90	141	12	CT	32	6
PANDEMIC_014	2021	302	26	1	47	113	23	CT	26	6
PANDEMIC_015	2021	345	21	1	115	103	10	CT	26	1
PANDEMIC_016	2021	414	19	3	30	127	35	CT	6	6
PANDEMIC_017	2021	353	18	3	79	95	10	CT	31	6
PANDEMIC_018	2021	30	22	2	68	371	NK	CT	16	3
PANDEMIC_019	2021	360	26	4	55	37	20	CT	62	6

PANDEMIC_020	2021	330	33	1	44	190	18	MRI	21	6
PANDEMIC_021	2021	500	38	1	65	350	8	MRI	26	6
PANDEMIC_022	2021	340	NK	1	40	40	20	CT	10	4
PANDEMIC_023	2021	340	20	1	46	42	10	MRI	30	4
PANDEMIC_024	2021	480	18	1	60	84	20	CT	48	4
PANDEMIC_025	2021	230	28	1	55	98	17	MRI	33	5
PANDEMIC_026	2021	440	35	1	45	26	20	MRI	68	4
PANDEMIC_027	2021	390	18	1	43	170	5	CT	13	3
PANDEMIC_028	2021	370	13	4	70	66	20	CT	21	6
PANDEMIC_029	2021	350	13,44	1		105	7	CT	20	6
PANDEMIC_030	2021	415	14	6	92	1	8	CT	19	6
PANDEMIC_031	2021	175	23	2	68	113	4	CT	13	6
PANDEMIC_032	2021	NK	12	2	58	145	79	CT	16	6
PANDEMIC_033	2021	NK	28	2	56	116	118	CT	12	6
PANDEMIC_034	2021	NK	13	1	46	182	3	CT	27	6

NK not known; *CRP* C-reactive protein; *INR* international normalized ratio, *aPTT* partial thromboplastin time; *mRS* modified Rankin Scale

1. Agarwal A, Vishnu VY, Vibha D, et al. Intracerebral Hemorrhage and SARS-CoV-2: Association or Causation. *Ann Indian Acad Neurol*. 2020;23(3):261-264. doi:10.4103/aian.AIAN_362_20

2. Al Saiegh F, Ghosh R, Leibold A, et al. Status of SARS-CoV-2 in cerebrospinal fluid of patients with COVID-19 and stroke. *J Neurol Neurosurg Psychiatry*. 2020;91(8):846-848. doi:10.1136/jnnp-2020-323522

3. Al-Dalahmah O, Thakur KT, Nordvig AS, et al. Neuronophagia and microglial nodules in a SARS-CoV-2 patient with cerebellar hemorrhage. *Acta Neuropathol Commun*. 2020;8(1):1-7. doi:10.1186/s40478-020-01024-2

4. Al-Olama M, Rashid A, Garozzo D. COVID-19-associated meningoencephalitis complicated with intracranial hemorrhage: a case report. *Acta Neurochir (Wien)*. 2020;162(7):1495-1499. doi:10.1007/s00701-020-04402-w

5. Benger M, Williams O, Siddiqui J, Sztriha L. Intracerebral haemorrhage and COVID-19: Clinical characteristics from a case series. *Brain Behav Immun*. 2020;88(June):940-944. doi:10.1016/j.bbi.2020.06.005

6. Cannac O, Martinez-Almoyna L, Hraiech S. Critical illness-associated cerebral microbleeds in COVID-19 acute respiratory distress syndrome. *Neurology*. 2020;95(11):498-499. doi:10.1212/WNL.0000000000010537

7. Carroll E, Lewis A. Catastrophic Intracranial Hemorrhage in Two Critically Ill Patients with COVID-19. *Neurocrit Care*. Published online May 2020:1-5. doi:10.1007/s12028-020-00993-5

8. Chougar L, Mathon B, Weiss N, Degos V, Shor N. Atypical Deep Cerebral Vein Thrombosis with Hemorrhagic Venous Infarction in a Patient Positive for COVID-19. *AJNR Am J Neuroradiol*. 2020;41(8):1377-1379. doi:10.3174/ajnr.A6642

9. Daci R, Kennelly M, Ferris A, et al. Bilateral Basal Ganglia Hemorrhage in a Patient with Confirmed COVID-19. *AJNR Am J Neuroradiol*. 2020;41(10):1797-1799. doi:10.3174/ajnr.A6712

10. Dakay K, Kaur G, Gulko E, et al. *Reversible Cerebral Vasoconstriction Syndrome and Dissection in the Setting of COVID-19 Infection.* Vol 29.; 2020. doi:10.1016/j.jstrokecerebrovasdis.2020.105011

11. De Stefano P, Nencha U, De Stefano L, Mégevand P, Seeck M. *Focal EEG Changes Indicating Critical Illness Associated Cerebral Microbleeds in a Covid-19 Patient.* Vol 5.; 2020. doi:10.1016/j.cnp.2020.05.004

12. Dixon L, Varley J, Gontsarova A, et al. COVID-19-related acute necrotizing encephalopathy with brain stem involvement in a patient with aplastic anemia. *Neurol Neuroimmunol Neuroinflammation*. 2020;7(5). doi:10.1212/NXI.0000000000000789

13. Franceschi AM, Ahmed O, Giliberto L, Castillo M. Hemorrhagic Posterior Reversible Encephalopathy Syndrome as a Manifestation of COVID-19 Infection. *AJNR Am J Neuroradiol*. 2020;41(7):1173-1176. doi:10.3174/ajnr.A6595

14. Ghani MU, Kumar M, Ghani U, Sonia F, Abbas SA. Intracranial hemorrhage complicating anticoagulant prophylactic therapy in three hospitalized COVID-19 patients. *J Neurovirol*. 2020;26(4):602-604. doi:10.1007/s13365-020-00869-6

15. Gogia B, Fang X, Rai P. Intracranial Hemorrhage in a Patient With COVID-19: Possible Explanations and Considerations. *Cureus*. 2020;12(8):e10159. doi:10.7759/cureus.10159

16. Gonçalves B, Righy C, Kurtz P. Thrombotic and Hemorrhagic Neurological Complications in Critically Ill COVID-19 Patients. *Neurocrit Care*. 2020;33(2):587-590. doi:10.1007/s12028-020-01078-z

17. Gupta NA, Lien C, Iv M. Critical illness-associated cerebral microbleeds in severe COVID-19 infection. *Clin Imaging*. 2020;68:239-241. doi:10.1016/j.clinimag.2020.08.029

18. Harrogate S, Mortimer A, Burrows L, Fiddes B, Thomas I, Rice CM. Non-aneurysmal subarachnoid haemorrhage in COVID-19. *Neuroradiology*. Published online August 2020:1-4. doi:10.1007/s00234-020-02535-4

19. Heman-Ackah SM, Su YRS, Spadola M, et al. Neurologically devastating intraparenchymal hemorrhage in COVID-19 patients on extracorporeal membrane oxygenation: A case series. *Neurosurgery*. 2020;87(2):E147-E151. doi:10.1093/neuros/nyaa198

20. Hemasian H, Ansari B. First case of Covid-19 presented with cerebral venous thrombosis: A rare and dreaded case. *Rev Neurol (Paris)*. 2020;176(6):521-523. doi:10.1016/j.neurol.2020.04.013

21. Hernandez-Fernandez F, Sandoval Valencia H, Barbella-Aponte RA, et al. Cerebrovascular disease in patients with COVID-19: Neuroimaging, histological and clinical description. *Brain*. 2020;143(10):3089-3103. doi:10.1093/brain/awaa239

22. Khattar NK, Sharma M, McCallum AP, et al. Intracranial hemorrhage in a young COVID-19 patient. *Interdiscip Neurosurg Adv Tech Case Manag*. 2020;22:100878. doi:10.1016/j.inat.2020.100878

23. Lévesque V, Millaire É, Corsilli D, Rioux-Massé B, Carrier F-M. Severe immune thrombocytopenic purpura in critical COVID-19. *Int J Hematol*. 2020;112(5):746-750. doi:10.1007/s12185-020-02931-9

24. Li J, Long X, Zhu C, et al. A case of COVID-19 pneumonia with cerebral hemorrhage. *Thromb Res*. 2020;193(May):22-24. doi:10.1016/j.thromres.2020.05.050

25. Montes-Ramirez J, Aquino-Lopez E. COVID-19-associated diffuse leukoencephalopathy and cerebral microbleeds during puerperium. *Int J Gynecol Obstet*. 2020;151(3):466-467. doi:10.1002/ijgo.13399

26. Motoie R, Akai M, Kitahara T, et al. Coronavirus Disease 2019 Complicated by Multiple Simultaneous Intracerebral Hemorrhages. *Intern Med Tokyo Jpn*. 2020;59(20):2597-2600. doi:10.2169/internalmedicine.5697-20

27. Muhammad S, Petridis A, Cornelius JF, Hänggi D. Letter to editor: Severe brain haemorrhage and concomitant COVID-19 Infection: A neurovascular complication of COVID-19. *Brain Behav Immun*. 2020;87:150-151. doi:10.1016/j.bbi.2020.05.015

28. Planinc D, El-Rekaby A, Sivakumar R, Saksena R, Ngeh J. Acute stroke showing cerebral infarcts and microbleeds in a 31-year-old man with covid-19 pneumonia. *Br J Hosp Med*. 2020;81(8):6-8. doi:10.12968/hmed.2020.0366

29. Shekhar R, Sheikh AB, Suriya SS, Upadhyay S, Zafar A. Neurological Complications Among Native Americans with COVID-19: Our Experience at a Tertiary Care Academic Hospital in the U.S. *J Stroke Cerebrovasc Dis Off J Natl Stroke Assoc*. 2020;29(12):105260. doi:10.1016/j.jstrokecerebrovasdis.2020.105260

30. Soldatelli MD, do Amaral LF, Veiga VC, Rojas SSO, Omar S, Marussi VHR. Neurovascular and perfusion imaging findings in coronavirus disease 2019: Case report and literature review. *Neuroradiol J*. 2020;33(5):368-373. doi:10.1177/1971400920941652

31. Thu SS, Matin N, Levine SR. Olfactory gyrus intracerebral hemorrhage in a patient with COVID-19 infection. *J Clin Neurosci*. 2020;79:275-276. doi:10.1016/j.jocn.2020.07.033

32. Usman AA, Han J, Acker A, et al. A Case Series of Devastating Intracranial Hemorrhage During Venovenous Extracorporeal Membrane Oxygenation for COVID-19. *J Cardiothorac Vasc Anesth*. 2020;34(11):3006-3012. doi:10.1053/j.jvca.2020.07.063

33. Vattoth S, Abdelhady M, Alsoub H, Own A, Elsotouhy A. Critical illness-associated cerebral microbleeds in COVID-19. *Neuroradiol J*. 2020;33(5):374-376. doi:10.1177/1971400920939229

34. Vu D, Ruggiero M, Choi WS, et al. Three unsuspected CT diagnoses of COVID-19. *Emerg Radiol*. 2020;27(3):229-232. doi:10.1007/s10140-020-01775-4

35. Wee NK, Fan EB, Lee KCH, Chia YW, Lim TCC. CT Fluid-blood levels in COVID-19 intracranial hemorrhage. *Am J Neuroradiol*. 2020;41(9):E76-E77. doi:10.3174/ajnr.A6672

36. Zulfiqar A-A, Lorenzo-Villalba N, Hassler P, Andrès E. Immune Thrombocytopenic Purpura in a Patient with Covid-19. *N Engl J Med*. 2020;382(18):e43. doi:10.1056/nejmc2010472

37. Sharifi-Razavi A, Karimi N, Rouhani N. COVID-19 and intracerebral haemorrhage: causative or coincidental? *New Microbes New Infect*. 2020;35. doi:10.1016/j.nmni.2020.100669

38. Rustemi O, Raneri F, Iannucci G, Volpin L, Segna A. Aneurysmal subarachnoid hemorrhage in a SARS-CoV-2 positive testing: casual or causal? *Br J Neurosurg*. 2020;0(0):1-2. doi:10.1080/02688697.2020.1787343

39. Batcik OE, Kanat A, Cankay TU, et al. COVID-19 infection produces subarachnoid hemorrhage; acting now to understand its cause: A short communication. *Clin Neurol Neurosurg*. Published online 2021:4.

40. Cezar-Junior AB, Faquini IV, Silva JLJ, et al. Subarachnoid hemorrhage and COVID-19. *Medicine (Baltimore)*. 2020;99(51):e23862. doi:10.1097/md.0000000000023862

41. Fayed I, Pivazyan G, Conte AG, Chang J, Mai JC. Intracranial hemorrhage in critically ill patients hospitalized for. *J Clin Neurosci*. Published online 2020:5.

42. Ghosh R, Dubey S, Kanti Ray B, Chatterjee S, Benito-León J. COVID-19 Presenting With Thalamic Hemorrhage Unmasking Moyamoya Angiopathy. *Can J Neurol Sci J Can Sci Neurol*. 2020;47(6):849-851. doi:10.1017/cjn.2020.117

43. Haroon KH, Patro SN, Hussain S, Zafar A, Muhammad A. Multiple Microbleeds: A Serious Neurological Manifestation in a Critically Ill COVID-19 Patient. *Case Rep Neurol*. 2020;3050:373-377. doi:10.1159/000512322

44. Mousa-Ibrahim F, Berg S, OdTPDetola O, Teitcher M, Ruland S. Intracranial Hemorrhage in Hospitalized SARS-CoV-2 Patients: A Case Series. :8.

45. Nicolas-Jilwan M, Almaghrabi RS. Diffuse necrotising leukoencephalopathy with microhaemorrhages in a patient with severe COVID-19 disease. *Neuroradiol J*. 2020;33(6):528-531. doi:10.1177/1971400920959324

46. Ordinola AAM, Osmar SS, Marussi VHR, et al. Hemorragia cerebral durante fase ativa de infecção por SARS-CoV-2 em paciente com angiopatia amiloide: relato de caso. *Rev Bras Ter Intensiva*. 2020;32(4):603-605. doi:10.5935/0103-507X.20200098

47. Pavlov V, Beylerli O, Gareev I, Torres Solis LF, Solís Herrera A, Aliev G. COVID-19-Related Intracerebral Hemorrhage. *Front Aging Neurosci*. 2020;12(October):1-6. doi:10.3389/fnagi.2020.600172

48. Tristán-Samaniego DP, Chiquete E, Treviño I, et al. COVID-19-related diffuse posthypoxic leukoencephalopathy and microbleeds masquerades as acute necrot. :6.

49. Daneshi SA, Taheri M, Fattahi A. SARS coronavirus 2 and central nervous system manifestations: causation, relation, or coexistence? a case series study and literature review. *Br J Neurosurg*. 2020;0(0):1-6. doi:10.1080/02688697.2020.1861433

50. Kirschenbaum D, Imbach LL, Rushing EJ, et al. Intracerebral endotheliitis and microbleeds are neuropathological features of COVID-19. *Neuropathol Appl Neurobiol*. 2020;(July):1-6. doi:10.1111/nan.12677

51. Mishra S, Choueka M, Wang Q, et al. Intracranial Hemorrhage in COVID-19 Patients. *J Stroke Cerebrovasc Dis Off J Natl Stroke Assoc*. 2021;30(4):105603. doi:10.1016/j.jstrokecerebrovasdis.2021.105603

52. Agarwal S, Jain R, Dogra S, et al. Cerebral Microbleeds and Leukoencephalopathy in Critically Ill Patients with COVID-19. *Stroke*. 2020;(September):2649-2655. doi:10.1161/STROKEAHA.120.030940

53. Altschul DJ, Unda SR, de La Garza Ramos R, et al. Hemorrhagic presentations of COVID-19: Risk factors for mortality. *Clin Neurol Neurosurg*. 2020;198:106112. doi:10.1016/j.clineuro.2020.106112

54. Coolen T, Lolli V, Sadeghi N, et al. Early postmortem brain MRI findings in COVID-19 non-survivors. *Neurology*. 2020;95(14):e2016-e2027. doi:10.1212/WNL.0000000000010116

55. D'Amore F, Vinacci G, Agosti E, et al. Pressing issues in COVID-19: Probable cause to seize SARSCoV-2 for Its preferential involvement of posterior circulation manifesting as severe posterior reversible encephalopathy syndrome and posterior strokes. *Am J Neuroradiol*. 2020;41(10):1800-1803. doi:10.3174/ajnr.A6679

56. Dogra S, Jain R, Cao M, et al. Hemorrhagic stroke and anticoagulation in COVID-19. *J Stroke Cerebrovasc Dis Off J Natl Stroke Assoc*. 2020;29(8):104984. doi:10.1016/j.jstrokecerebrovasdis.2020.104984

57. Fitsiori A, Pugin D, Thieffry C, Lalive P, Vargas MI. COVID-19 is Associated with an Unusual Pattern of Brain Microbleeds in Critically Ill Patients. *J Neuroimaging Off J Am Soc Neuroimaging*. 2020;30(5):593-597. doi:10.1111/jon.12755

58. Katz JM, Libman RB, Wang JJ, et al. COVID-19 Severity and Stroke: Correlation of Imaging and Laboratory Markers. *AJNR Am J Neuroradiol*. Published online October 2020. doi:10.3174/ajnr.A6920

59. Lin E, Lantos JE, Strauss SB, et al. Brain Imaging of Patients with COVID-19: Findings at an Academic Institution during the Height of the Outbreak in New York City. *AJNR Am J Neuroradiol*. Published online August 2020. doi:10.3174/ajnr.A6793

60. Masur J, Freeman CW, Mohan S. A double-edged sword: Neurologic complications and mortality in extracorporeal membrane oxygenation therapy for COVID-19-related severe acute respiratory distress syndrome at a tertiary care center. *Am J Neuroradiol*. 2020;41(11):2009-2011. doi:10.3174/ajnr.A6728

61. Nawabi J, Morotti A, Wildgruber M, et al. Clinical and Imaging Characteristics in Patients with SARS-CoV-2 Infection and Acute Intracranial Hemorrhage. *J Clin Med*. 2020;9(8):2543. doi:10.3390/jcm9082543

62. Radmanesh A, Derman A, Lui YW, et al. COVID-19-associated Diffuse Leukoencephalopathy and Microhemorrhages. *Radiology*. 2020;297(1):E223-E227. doi:10.1148/radiol.2020202040

63. Requena M, Olivé-Gadea M, Muchada M, et al. COVID-19 and Stroke: Incidence and Etiological Description in a High-Volume Center. *J Stroke Cerebrovasc Dis Off J Natl Stroke Assoc*. 2020;29(11):105225. doi:10.1016/j.jstrokecerebrovasdis.2020.105225

64. Rothstein A, Oldridge O, Schwennesen H, Do D, Cucchiara BL. Acute Cerebrovascular Events in Hospitalized COVID-19 Patients. *Stroke*. 2020;(September):219-222. doi:10.1161/STROKEAHA.120.030995

65. Sawlani V, Scotton S, Nader K, et al. COVID-19-related intracranial imaging findings: a large single-centre experience. *Clin Radiol*. 2021;76(2):108-116. doi:10.1016/j.crad.2020.09.002

66. Siegler JE, Cardona P, Arenillas JF, et al. Cerebrovascular events and outcomes in hospitalized patients with COVID-19: The SVIN COVID-19 Multinational Registry. *Int J Stroke Off J Int Stroke Soc*. Published online September 2020:1747493020959216. doi:10.1177/1747493020959216

67. Trifan G, Goldenberg FD, Caprio FZ, et al. Characteristics of a Diverse Cohort of Stroke Patients with SARS-CoV-2 and Outcome by Sex. *J Stroke Cerebrovasc Dis Off J Natl Stroke Assoc*. 2020;29(11):105314. doi:10.1016/j.jstrokecerebrovasdis.2020.105314

68. Sweid A, Hammoud B, Bekelis K, et al. Cerebral ischemic and hemorrhagic complications of coronavirus disease 2019. *Int J Stroke*. 2020;15(7):733-742. doi:10.1177/1747493020937189

69. Bermea RS, Raz Y, Sertic F, et al. Increased Intracranial Hemorrhage Amid Elevated Inflammatory Markers in those with COVID-19 Supported with Extracorporeal Membrane Oxygenation. *Shock*. 2021;Publish Ahead of Print. doi:10.1097/SHK.0000000000001730

70. Büttner L, Bauknecht HC, Fleckenstein FN, et al. Neuroimaging Findings in Conjunction with Severe COVID-19. *RoFo Fortschritte Auf Dem Geb Rontgenstrahlen Bildgeb Verfahr*. Published online 2021. doi:10.1055/a-1345-9784

71. Dixon L, Mcnamara C, Gaur P, et al. Cerebral microhaemorrhage in COVID-19: A critical illness related phenomenon? *Stroke Vasc Neurol*. 2020;5(4):315-322. doi:10.1136/svn-2020-000652

72. John S, Hussain SI, Piechowski-Jozwiak B, et al. Clinical characteristics and admission patterns of stroke patients during the COVID 19 pandemic: A single center retrospective, observational study from the Abu Dhabi, United Arab Emirates. *Clin Neurol Neurosurg*. 2020;199(January). doi:10.1016/j.clineuro.2020.106227

73. Lang CN, Dettinger JS, Berchtold-Herz M, et al. Intracerebral Hemorrhage in COVID-19 Patients with Pulmonary Failure: A Propensity Score-Matched Registry Study. *Neurocrit Care*. Published online 2021. doi:10.1007/s12028-021-01202-7

74. Lawton MT, Alimohammadi E, Bagheri SR, et al. Coronavirus disease 2019 (COVID-19) can predispose young to Intracerebral hemorrhage: a retrospective observational study. *BMC Neurol*. 2021;21(1):83. doi:10.1186/s12883-021-02109-8

75. Lersy F, Willaume T, Brisset JC, et al. Critical illness-associated cerebral microbleeds for patients with severe COVID-19: etiologic hypotheses. *J Neurol*. 2020;(0123456789). doi:10.1007/s00415-020-10313-8

76. Shahjouei S, Naderi S, Li J, et al. Risk of stroke in hospitalized SARS-CoV-2 infected patients: A multinational study. *EBioMedicine*. 2020;59. doi:10.1016/j.ebiom.2020.102939
